# Perceptions and practices of aged care pharmacists regarding osteoporosis management: a qualitative study

**DOI:** 10.1007/s11096-023-01586-w

**Published:** 2023-05-10

**Authors:** Catherine Laird, Kylie A. Williams, Helen Benson

**Affiliations:** Graduate School of Health, University of Technology Sydney, PO Box 123, Sydney, NSW 2007 Australia

**Keywords:** Aged care, Interdisciplinary collaboration, Medication management, Osteoporosis, Pharmacist

## Abstract

**Background:**

Osteoporosis is a common but sub-optimally managed disease among aged care residents. Although pharmacists are one of the key healthcare providers responsible for osteoporosis medication management there is limited research on their involvement.

**Aim:**

This study explored the perceptions and practices of Australian pharmacists regarding osteoporosis management for aged care residents.

**Method:**

Semi-structured interviews were conducted with aged care pharmacists. Interviews were recorded, transcribed, and analysed using a constructivist grounded theory approach.

**Results:**

Twenty-one aged care pharmacists were interviewed. Three main themes were identified: osteoporosis is highly prevalent but given low priority, factors affecting pharmacists’ management of osteoporosis, and optimism for the future role of pharmacists in osteoporosis management. The complexity of aged care residents’ healthcare needs and the silent, insidious nature of osteoporosis contribute to the low priority it is afforded. Barriers identified by pharmacists included their current practice model, limited access to residents’ medical histories and difficulties accessing bone mineral density (BMD) testing. Interdisciplinary collaboration and education regarding osteoporosis management were seen as facilitators. Pharmacists were optimistic that an embedded practice model would improve their capacity to influence osteoporosis management.

**Conclusion:**

The high prevalence and low priority of osteoporosis in the aged care setting presents pharmacists with an opportunity to improve medication management and reduce fracture risk. Barriers to osteoporosis management identified by pharmacists can be addressed by interdisciplinary collaboration and education. Pharmacists being embedded in aged care could enable more opportunities to contribute to the interdisciplinary team and become champions of osteoporosis management.

## Impact statements

- Improving interdisciplinary collaboration and raising awareness of osteoporosis are the proposed mechanisms to enhance the involvement of pharmacists in optimising osteoporosis management for aged care residents.
- Embedding pharmacists in aged care facilities offers them an opportunity to become the champions of osteoporosis management for aged care residents.

## Introduction

Osteoporosis is a disease in which the quality and density of bone is reduced, increasing susceptibility to fracture [1]. Osteoporosis is predominantly a disease impacting the elderly [1]. The globally aging population is anticipated to result in an escalation in osteoporosis-related fractures, specifically the worldwide incidence of osteoporotic hip fractures is projected to increase from 1.66 million in 1990 to 6.26 million by 2050 [2]. Over the last thirty years, available pharmacological therapies for osteoporosis have expanded, including the introduction of the antiresorptive therapies bisphosphonates and denosumab [1]. Although diagnostic criteria and appropriate treatments are readily available, worldwide osteoporosis is underdiagnosed and undertreated [1, 3].

Residents of aged care facilities are particularly susceptible to osteoporosis [4, 5]. International studies indicate that more than 80% of aged care residents have osteoporosis and they experience a disproportionate number of fractures compared to the general population [6–8]. Although osteoporosis therapies effectively prevent fractures in aged care residents, Australian and international studies have shown that undertreatment is widespread and worsening [5, 9, 10]. Although the reasons for this are unclear, it has been postulated the decline is due to reports of rare adverse effects [5, 9, 10].

Aged care residents are reported to have a greater level of medical complexity than community-dwelling individuals, which complicates osteoporosis management [10–12]. Aged care residents are usually frail, have multiple comorbidities, and take multiple medications [11, 12]. These factors make aged care residents highly susceptible to adverse drug reactions [12]. In Australia, most aged care residents die in care with a median length of stay of less than two years [13]. This limited life expectancy of residents has been attributed to aged care residents having different goals of care to community-dwelling individuals [14–16].

Within aged care facilities physicians, nurses, and pharmacists are the healthcare providers primarily responsible for medication management [15]. Previous studies have reviewed the awareness of physicians and nurses regarding osteoporosis [17–21]. These studies identified knowledge gaps that impede management.

There is growing interest in the role of pharmacists in improving the medication management of aged care residents [15, 22, 23]. In recent years, Australia has followed the global trend of pharmacy practice evolving from its traditional dispense and supply focus to an emphasis on non-dispensing services [22–24]. The provision of these services for aged care residents has been shown to improve medication appropriateness [23].

In Australia, non-dispensing pharmacy services for residents of aged care are funded by two national programmes: the Residential Medication Management Review (RMMR) program and the Quality Use of Medicines (QUM) program [25, 26]. The RMMR program is akin to “clinical medication reviews” in the UK, “comprehensive medication reviews” in the US, and “MedsCheck LTC” in Canada [22, 27]. Australian pharmacists undertake additional training to be accredited to conduct medication reviews [25]. Upon receiving a referral from the resident’s physician, an accredited pharmacist completes a comprehensive medication review and provides a written report to the referrer [25]. The physician then considers this report, implementing recommendations at their discretion [25]. The QUM program aims to improve medication management at a facility-wide level [26]. This program involves activities such as staff education, continuous improvement activities, and participation in medication advisory committees (MAC) [26]. Both RMMRs and QUM services are completed by pharmacists practising in an external visiting capacity [25, 26]. In an effort to improve aged care residents’ medication management, in 2022, the Australian government announced funding to enable pharmacists to transition from the current visiting practice model to being embedded in aged care facilities [28].

A recent systematic literature review found that pharmacists undertaking non-dispensing services can improve osteoporosis management [29]. Utilising these services, pharmacists increased osteoporosis investigation, treatment commencement and adherence [29]. However, this literature review identified that there is limited evidence regarding pharmacist interventions for osteoporosis in the aged care setting [29]. It is theorised that pharmacists undertaking non-dispensing services can improve osteoporosis management for aged care residents.

### Aim

This study aimed to explore the perceptions and practices of Australian pharmacists regarding osteoporosis management for aged care residents.

### Ethics approval

The study was approved by the Human Research Ethics Committee (HREC) at the University of Technology Sydney (ETH22-7101). All pharmacists provided written informed consent.

## Method

The study has been reported per the consolidated criteria for reporting qualitative research (COREQ) [30].

### Study design

A constructivist grounded theory approach, as developed by Charmaz, was utilised for this study [31]. Grounded theory is an inductive research method that lends itself to studying people’s experiences in areas where previous research is limited [32]. Constructivist grounded theory is a highly reflexive approach to grounded theory suitable for use by researchers with pre-existing knowledge of the area being investigated [31].

A single semi-structured interview was conducted with each pharmacist utilising online video conferencing platforms (Zoom™ and Microsoft Teams™). Individual interviews were used rather than focus groups as they encouraged frank discussion, avoided social desirability bias and assured participants of confidentiality [32].

### Study setting and sampling

The study involved pharmacists from across Australia who provide non-dispensing services to aged care facilities and their residents. Initially, two methods of sampling were employed by the research team. Purposive sampling involved approaching pharmacists directly via email utilising publicly available contact details and advertisement of the study through professional bodies and relevant social media groups. Additionally, snowball sampling was implemented whereby participating pharmacists were asked to share contact details of willing colleagues who were then contacted by email. Pharmacists were provided with a participant information sheet to allow them to make an informed decision to participate. These approaches were conducive to theoretical sampling as the study progressed. Theoretical sampling, a core element of grounded theory, involves participants being selected to assist researchers in testing and refining the emerging theory [31]. Recruitment occurred in July and August 2022.

### Data collection

One researcher (CL) was responsible for conducting interviews, which were audio-recorded and transcribed verbatim both manually and using the auto-transcribe function of online video conferencing platforms. The resulting transcripts were cross checked for accuracy and returned to participants. Field notes were made during interviews.

**Fig. 1:**
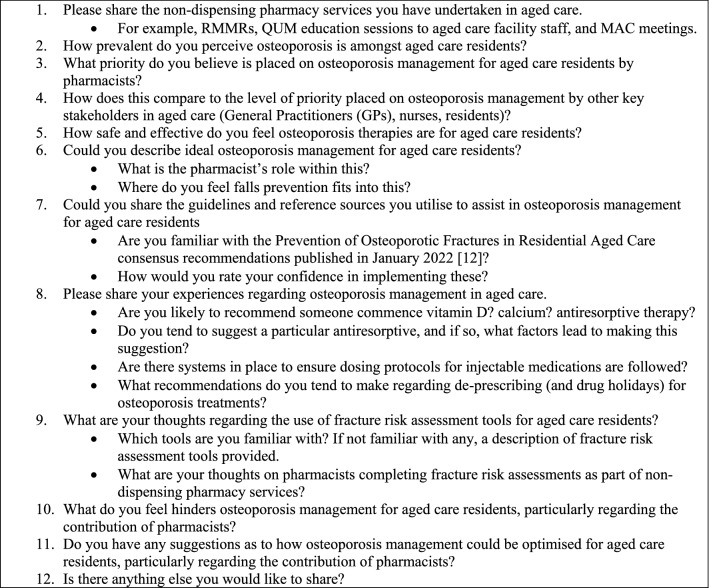
Interview topic guide and prompts

The interview guide (see Fig. 1) was formulated using established practices on interview development, including piloting of the interview guide [33, 34]. The guide focused on pharmacists’ practices and perceptions regarding osteoporosis management.

### Data analysis

In accordance with constructivist grounded theory, data collection and analysis occurred simultaneously. Interviews and analysis occurring in a cyclical nature until theoretical saturation was achieved and then confirmed by undertaking a further three interviews. Theoretical saturation describes the point where new date does not result in additional properties of a category nor further insights about the theory [31].

De-identified transcripts were imported into NVivo 12 Pro™ to facilitate analysis. Coding was undertaken by one researcher (CL) and reviewed for accuracy by a second researcher (HB). Coding occurred in three stages: initial, focused, and theoretical. Constant comparative analysis was used to identify and refine emerging themes. Regular meetings were held with by the three researchers to reflect upon the findings and resolve any disagreements on data analysis.

### Reflexivity

Reflexivity, recognising the researcher’s presence in the research, is an essential element of constructivist grounded theory [31]. The research team consisted of three Australian pharmacists. The researcher (CL), who conducted the interviews, has experience providing non-dispensing pharmacy services for aged care residents and is a doctoral candidate. Prior to undertaking interviews, CL completed university modules in qualitative research. Participants were advised of the interviewer’s background before the interviews. Five participants were previously known to CL through professional associations. All participants were advised that the interviewer had assumed the role of a neutral researcher. The employment of open line-by-line coding for the initial coding of all transcripts limited potential bias resulting from the researcher’s background, as this method prevents the researcher from inputting their own beliefs on the data [31].

## Results

Semi-structured interviews were conducted with twenty-one pharmacists during July and August 2022. Interviews lasted between 20 and 46 min (mean 28.75 min, SD 5.35 min). As shown in Table 1, the participating pharmacists were representative of a mix of age ranges and professional experience in aged care. Further, pharmacists were representative of metropolitan and rural practice locations from four of the six states of Australia. One pharmacist was employed as an embedded pharmacist at an aged care facility. The remainder performed non-dispensing pharmacy services in an external visiting role. When directly quoting participants they are referred to by their participant number, gender, number of years servicing aged care facilities and current role.

**Table 1:** Pharmacist characteristics

Gender	Female	16
	Male	5
Age	25–34	2
	35–44	10
	45–54	3
	55–64	5
	≥ 65	1
State	New South Wales	5
	Queensland	11
	South Australia	2
	Victoria	3
Location of aged care facilities serviced	Metropolitan	6
	Rural	6
	Both metropolitan and rural	9
Number of years servicing aged care facilities	< 5	1
	5–9	4
	10–14	2
	15–19	8
	≥ 20	6
Current role	External visiting pharmacist	20
	Embedded pharmacist	1

Data analysis identified three major themes, as described in Table 2.

**Table 2:** Themes and subthemes

Themes	Subthemes
Osteoporosis is highly prevalent but given low priority	Silent disease
	Competing health care needs of residents
Factors affecting pharmacists’ management of osteoporosis	Barriers
	Current aged care pharmacist practice model
	Limited access to residents’ medical histories
	Difficulty accessing bone mineral density (BMD) testing
	Facilitators
	Interdisciplinary collaboration
	Education
Optimism for the future role of pharmacists in osteoporosis management	Embedded pharmacists will have the capacity to address osteoporosis

### Theme 1: osteoporosis is highly prevalent but given low priority

Most pharmacists reported osteoporosis is highly prevalent amongst aged care resident however, it is afforded a low level of priority. Two sub-themes to explain this low prioritisation were found.

#### Silent disease

Pharmacists frequently commented on the insidious nature of osteoporosis, describing it as:

“…*a bit of a silent in the background thing…*” (P10; Female (F); 5–9; visiting pharmacist).

Pharmacists felt that the silent nature of osteoporosis was associated with both underdiagnosis and low priority in residents with a diagnosis:

“…*unless of course…they’ve had a fall and a fracture and all of a sudden it comes… up the priority list. But certainly, speaking if they’ve just got a diagnosis … it does get left*.” (P8; F; 15–19; visiting pharmacist).

#### Complex healthcare needs of residents

All participating pharmacists recognised that aged care residents have complex healthcare needs associated with their frailty, multiple morbidities and use of multiple medications. As a result, of these complex healthcare needs, osteoporosis was reported to be overshadowed by:

“*…so many other disease states that seem to take priority*” (P4; F; ≥ 20; visiting pharmacist).

Pharmacists reported that the complex healthcare needs of residents impact the recommendations they make regarding osteoporosis management. In particular, pharmacists placed a high emphasis on deprescribing, citing the negative health outcomes associated with polypharmacy in this population. This is illustrated by one pharmacist's comment:

“…*in an aged care facility the first thing I think about is deprescribing*” (P1; Male (M); 15–19; visiting pharmacist).

Pharmacists discussed osteoporosis treatments in two categories, nutritional supplements and antiresorptive therapies. The nutritional supplements, calcium and vitamin D were reported to be frequently used by aged care residents. Pharmacists unanimously agreed that vitamin D should be supplemented for all residents receiving antiresorptive therapy. However, views on calcium supplements and the universal use of vitamin D for aged care residents were divided. These nutritional supplements were frequently seen as deprescribing opportunities:

*“…the big, huge things right now are psychotropics and polypharmacy; and … vitamin D and calcium…are the things people go ‘we can get rid of that’ because we need to get the numbers down…”* (P18; F; 10–14; visiting pharmacist).

Pharmacists were unlikely to recommend the commencement of antiresorptive therapies. Whilst pharmacists viewed antiresorptive therapies to be effective in the general population, they were hesitant to recommend them for aged care residents due to concerns of contributing to polypharmacy coupled with the uncertainty of the clinical benefit of antiresorptive therapies being realised given the limited life expectancy of residents:

*“it sounds awful, but these people…are approaching end of life. Is it [osteoporosis] something that's…worth treating or is it not?*” (P4; F; ≥ 20; visiting pharmacist).

### Theme 2: factors affecting pharmacists’ management of osteoporosis

Pharmacists identified both barriers and facilitators to osteoporosis management.

#### Barriers

Three barriers to osteoporosis management of aged care residents were identified: the current aged care pharmacist practice model, limited access to residents’ medical histories, and difficulty accessing bone mineral density (BMD) testing.

Pharmacists viewed the current external visiting practice model, which provides episodic limited care centering around the RMMR and QUM programs, to be a barrier to their involvement in osteoporosis management.

Medication reviews were seen as a highly valuable service by pharmacists, however shortcomings of the RMMR programme were identified which limit its impact on osteoporosis management. Pharmacists explained that the timing of reviews is frequently determined by their visiting schedule rather than clinical need. Pharmacists also identified that the provision of reports to convey information, without clinical discussion with the physician, could impede review outcomes:

“*the way we deliver the information through a written report, I don’t think is the best way to do things for two reasons: you can be a lot more convincing, and you can be a lot more flexible in a discussion*” (P7; M; 5–9; embedded pharmacist).

Pharmacists believed non-dispensing pharmacy services, in addition to medication reviews, could improve osteoporosis management. Theoretically, these would occur as part of the QUM program; however, pharmacists reported that funding for this program restricts pharmacists from undertaking such services. Currently, a minimal fixed payment rate (equivalent to approximately two hours of a pharmacist’s time per quarter) is paid for providing the mandated QUM services [26]. While the provision of additional services is encouraged, no further funding is available for this:

*“…I think it can be way better done if it's part of a QUM activity. But by and large, I see and have experienced pharmacists not spending as much time on QUM because of the flat rate funding”* (P2; F; 15–19 years; visiting pharmacist).

Accessing the resident’s medical history can be difficult and impede management. Pharmacists reported that at the time of admission to the facilities, residents are frequently transferred to the care of a new physician with minimal medical records. Participating pharmacists explained they frequently need to compile records from a variety of sources, including previous physicians, hospitals and community pharmacies records, to determine resident’s medical histories. One pharmacist explained:

“…*previous fractures, previous use of antiresorptive drugs, previous diagnosis of osteoporosis, all of those things can be quite tricky to find…”* (P7; M; 5–9; embedded pharmacist).

Difficulties accessing medical history were linked to both undertreatment and overtreatment of osteoporosis. All pharmacists identified the potential for denosumab doses to be missed or delayed, many recounting their difficulties in determining when doses are due:

“*Every time I see someone with Prolia® [denosumab] on the medication chart, I dig as to when they last had it; half the time, it isn't clear”* (P8; F; 15–19; visiting pharmacist).

Further, a lack of information can prevent pharmacists from making recommendations concerning bisphosphonate drug holidays:

*“…we usually don't know how long they've been on it before coming into the facility, and their stay usually isn't long enough for just our episode of care to reach that milestone for drug holidays”* (P6; M; 5–9; visiting pharmacist).

All pharmacists reported that accessing BMD testing is difficult for aged care residents, which impedes diagnosis and monitoring of therapy effectiveness. The logistical challenges involved in transporting residents off-site for BMD testing can make it impractical:

*“…the difficulties in aged care are that people have other conditions like dementia and osteoarthritis and reasons why they can't really have BMD done very often or at all. It can be too distressing for them to go for bone mineral density testing”* (P11; F; ≥ 20; visiting pharmacist).

#### Facilitators

Two facilitators for osteoporosis management of aged care residents were identified: interdisciplinary collaboration and education.

All pharmacists commented that osteoporosis management could be improved through interdisciplinary collaboration. Osteoporosis management was seen to be multifaceted with falls risk reduction being a crucial component of fracture prevention. Along with deprescribing falls risk-inducing drugs, pharmacists placed a high emphasis on balance and strength conditioning to reduce falls risk. Consequently, a multidisciplinary team was deemed necessary to achieve optimal osteoporosis management:

*“…there must be a GP on board and if you've got a physio and a nurse and an OT and a pharmacist, I think you're going to optimise how osteoporosis is managed. So, I think it absolutely needs to be multidisciplinary”* (P2; F; 15–19; visiting pharmacist).

Education on osteoporosis management for all aged care stakeholders was viewed by most pharmacists as a facilitator. This is illustrated by one pharmacist’s response when asked for suggestions on how osteoporosis management could be improved:

*“…educating the staff about osteoporosis management and educating the families as well…”* (P15; F; < 5; visiting pharmacist).

Many pharmacists acknowledged a need to increase their own knowledge of osteoporosis management for aged care residents. Although an Australian consensus statement for osteoporosis management of aged care residents has been available (and periodically revised) for over 12 years, awareness of this was found to be low [14]. A typical response when pharmacists were asked if they were familiar with this statement was:

*“Nope, and that is a problem because we should know about it”* (P12; F; ≥ 20; visiting pharmacist).

### Theme 3: optimism for the future role of pharmacists in osteoporosis management

Pharmacists were optimistic that the impending practice model change would enable them to increase their impact on osteoporosis management. The proposed change will replace the current external visiting model with one where pharmacists are permanently embedded in aged care facilities.

#### Embedded pharmacists will have the capacity to address osteoporosis

All pharmacists believed that an embedded practice model would give them greater capacity to address osteoporosis:

*“…definitely there's more things I would pursue, but time and … what we get paid for reviews, you’re not really able to do some of the stuff you would like …. So, I do think, there is the potential there to have this [osteoporosis] treated better in aged care, through this new program”* (P17; F; 15–19; visiting pharmacist).

Most pharmacists welcomed the concept of an aged care resident-specific fracture risk assessment tool to guide management. Pharmacists believed completing such risk assessments would be a task they could undertake when embedded in aged care facilities:

*“I think, running through some of those risk calculators, would be an excellent job for a pharmacist to do”* (P6; M; 5–9; visiting pharmacist).

Several pharmacists identified that embedded pharmacists could become the champions of osteoporosis management within aged care, sentiments supported by the one pharmacist currently employed in this role.

*“I think pharmacists could actually be a driver because … although it's not total management, a lot of the management of osteoporosis prevention is drug therapy. So that really should be driven by the pharmacists”* (P19; F; 15–19; visiting pharmacist).

## Discussion

### Statement of key findings

This study established three major themes relevant to aged care pharmacists’ perceptions and practices regarding osteoporosis management: osteoporosis is highly prevalent but given low priority, factors affecting pharmacists’ management of osteoporosis, and optimism for the future role of pharmacists.

Pharmacists were aware that osteoporosis is highly prevalent among the aged care population. Although osteoporosis related fractures have significant clinical outcomes for residents, pharmacists reported that osteoporosis management is given low priority. This low priority was attributed to the silent nature of osteoporosis coupled with the complex healthcare needs of residents, including an emphasis on reducing polypharmacy.

Pharmacists identified barriers and facilitators to the effective management of osteoporosis. Pharmacists reported that the current external visiting practice model impedes their involvement in osteoporosis management. Limited access to residents’ medical histories contributed to both undertreatment and overtreatment, while difficulties accessing BMD testing were linked to underdiagnosis and lack of monitoring. Pharmacists identified interdisciplinary models of care and increased education as facilitators of osteoporosis management. Pharmacists were optimistic that an impending practice model change, which will see pharmacists embedded in aged care facilities, will enable them to play a greater role in osteoporosis management.

### Strengths and weaknesses

This study is the first to explore the perspectives and practices of aged care pharmacists regarding osteoporosis management. The sampling methods employed ensured that participating pharmacists were representative of a broad mix of Australian practice locations and had varying levels of professional experience. Consequently, a major strength of this study is that it provides a broad overview of the perspectives and practices of aged care pharmacists regarding osteoporosis management in Australia.

The interviewer’s experience as an aged care pharmacist facilitated an immediate connection with the pharmacists interviewed. It is believed this enabled a high level of trust and frankness during interviews. However, it is recognised that the personal experience of the interviewer could introduce an element of bias. The use of line-by-line coding and review of coding for accuracy by a second researcher ensured that codes and subsequent themes were developed from the data [31]. The accuracy of transcripts was enhanced by the use of two means of transcription and returning transcripts to participants.

### Interpretation

The perceptions of pharmacists regarding osteoporosis closely resemble those previously reported among physicians and nurses [17–21]. Notably, Salminen, Piispanen and Toth-Pal, in their qualitative study on physicians’ views of osteoporosis management, identified the main theme

*“Osteoporosis- a silent disease overshadowed by other conditions*” [21].

Pharmacists placed a high emphasis on avoiding the use of polypharmacy for aged care residents due to the association of negative health outcomes with polypharmacy [12]. Despite Australian and international guidelines advocating universal use of vitamin D supplementation by aged care residents, pharmacists (as with studies involving physicians and nurses) held mixed views on its use, with many viewing vitamin D as a deprescribing opportunity to reduce polypharmacy [14, 17, 35, 36]. Similarly, pharmacists were hesitant to recommend the commencement of antiresorptive therapies, citing avoiding polypharmacy along with uncertainties around the effectiveness of these agents in aged care residents given their limited life expectancy (concerns also shared by physicians and nurses) [17, 18, 35]. Pharmacists believed education on osteoporosis management for all stakeholders in aged care would assist in guiding recommendations as to when osteoporosis treatment is indicated. This view aligns with studies involving physicians and nurses, which have identified the need for education on osteoporosis management specific to aged care residents [17, 18].

Difficulty accessing BMD testing was identified by pharmacists in this study as a barrier to management. Consistent with published literature, logistical challenges were reported to prohibit aged care residents from undergoing BMD testing [16, 17, 37]. This, combined with the high prevalence of osteoporosis in aged care residents, has led to questioning the clinical usefulness of BMD testing for aged care residents [16, 37]. There is literature to support that evaluation for osteoporosis therapy in this population should be based on clinical factors of high fracture risk rather than BMD testing [16, 37]. Consequently, aged care resident-specific fracture risk assessment tools to guide management have been developed [16, 37]. Completing fracture risk assessment tools and participating in case conferences that enable clinical discussion are examples of a non-dispensing service embedded pharmacists could undertake as part of an interdisciplinary team to improve osteoporosis management [29].

### Further research

Osteoporosis management has been described as a “Bermuda triangle” of healthcare professionals, into which patients with fragility fractures disappear [21]. When the results of this study are considered along with those involving physicians and nurses, there is a clear need for a proactive collaborative approach if improvements in osteoporosis management for aged care residents are to materialise. Physicians have previously expressed a desire to share the responsibility of osteoporosis management, suggesting that others could take on the coordination of osteoporosis management and complete screening [21]. This study has demonstrated that aged care pharmacists are willing to fulfil this role, and an embedded practice model could allow them to do so. To enable pharmacists to achieve this, developing clinical support resources incorporating an aged care specific fracture risk assessment tool is recommended. Further research on implementing a sustainable pharmacist-led collaborative osteoporosis management program and evaluating the impact of the embedded pharmacist practice model on osteoporosis management is recommended.

## Conclusion

The high prevalence and low priority given to osteoporosis in the aged care setting is an opportunity for pharmacists to improve medication management and reduce fracture risk. The current visiting practice model, along with limited access to residents’ medical histories and difficulty accessing BMD testing, are seen as barriers to osteoporosis management by pharmacists. These barriers could be addressed by interdisciplinary collaboration and increased osteoporosis education for healthcare staff. Pharmacists being embedded in aged care facilities will give them greater opportunity to be part of the multidisciplinary team and enable them to become the champions of osteoporosis management in aged care.
